# Complex Posterior Glenohumeral Instability Case Management

**DOI:** 10.7759/cureus.24198

**Published:** 2022-04-17

**Authors:** Rony H Melhem, Eliane K Doumith, Marc Soubeyrand

**Affiliations:** 1 Orthopedic Surgery, Dr. Sulaiman Al-Habib Hospital, Riyadh, SAU; 2 Radiology, Lebanese American University (LAU) Medical Center-Rizk Hospital, Beirut, LBN; 3 Orthopedics Department, Bicêtre Hospital, AP-HP, Paris, FRA

**Keywords:** labrum, coracohumeral ligament, surgical technique, glenohumeral joint, posterior shoulder instability

## Abstract

A 21-year-old male patient suffering from insidious shoulder instability from neurogenic and structural attenuation of shoulder stabilizer, due to old minor cerebrovascular accident, presented with a two-year history of repetitive shoulder dislocation, operated by fixing the biceps tendon to its insertion on the superior labrum, correction of the glenoid version and reattaching the subscapular tendon along with a new technique for stabilization of the shoulder replicating the coracohumeral ligament with a ligament advanced reinforcement system (LARS) transplant. Following structured physical therapy, our patient returned to normal daily activities at 15 months.

## Introduction

Shoulder instability is an excessive translation of the humeral head in relation to the glenoid [1]. Symptoms usually range from a simple discomfort feeling of shoulder instability to severe pain and complete loss of motion with frank dislocation. Posterior instability may occur over a large spectrum, from subtle posterior subluxation causing minimal symptoms to frank posterior dislocation [2].

Several injury mechanisms may be responsible for posterior instability of the shoulder, beginning with acute traumatic posteriorly directed force resulting in capsulolabral detachment, second repetitive microtrauma also leading to attenuation of the posterior capsule, and finally, insidious onset laxity resulting in stretching of the posterior capsule and passive stabilizers.

In this study, we present a 21-year-old male patient suffering from insidious shoulder instability from neurogenic and structural attenuation of shoulder stabilizer due to an old minor cerebrovascular accident, with a two-year history of repetitive shoulder dislocation.

After the failure of our previous interventions (fixing the biceps tendon superior labrum from anterior to posterior (SLAP) lesion and correction of glenoid version) to stabilize the shoulder, one month later we attempted a new surgical technique for shoulder stabilization by replicating the coracohumeral ligament with a ligament advanced reinforcement system (LARS), passing it through a tunnel drilled in the humeral head.

The patient was informed that data concerning the case could be submitted for publication, and he provided consent.

## Case presentation

A right-hand-dominant 21-year-old patient consulted two years ago for a marked feeling of shoulder instability associated with multiple episodes of non-painful right posterior shoulder dislocation; on physical exam, he had competent deltoid muscle, shoulder abduction of 90°, external and internal rotation of 45 degrees each, negative Belly Press test, supraspinatus muscle was very weak, and there was no amyotrophy in the supra and infraspinatus muscles. The shoulder could be easily dislocated and reduced (Video 1).

**Video 1:** Video showing repetitive posterior dislocation and reduction of the shoulder joint

The patient was seen again after one year, consulting for the same complaint. He mentioned a marked feeling of instability with associated pain requiring occasional use of pain medications and a relatively limited shoulder function during daily activities.

The constant score, including objective and subjective shoulder assessment, was around 40 percent. The patient’s estimation of the value of the affected shoulder as a percentage of that of an entirely normal shoulder amounts to 40% [3].

On physical exam, he had shoulder abduction of 90° and external rotation limited to 45 degrees. Jerk test, Kim test, posterior load, and shift test were positive (these tests are highly indicative of posterior instability) [4-5]. He also had a very weak subscapularis muscle graded 1 out of 5, functional pectoralis major, and diffuse dysesthesia of the right upper limb with elbow stiffness. The Beighton score, used to assess generalized ligamentous laxity, which has been shown to correlate with shoulder instability, was zero [6].

There were more than 10 episodes of dislocation daily; besides the shoulder dislocation, the patient had multiple comorbidities like steroid-induced osteoporosis, homozygous sickle cell anemia, cerebrovascular accident in 2004 with residual right hemiparesis (muscle power in right upper limb graded 4 out of 5) under physical therapy, and multiples episodes of cytomegalovirus (CMV) reactivation.

**Figure 1:**
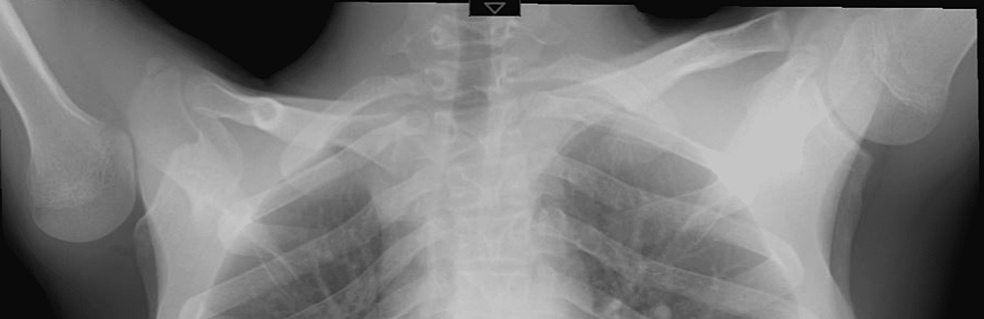
Standard X-ray showing an absence of congruence of the glenohumeral joint upon abduction of the arm

Standard X-ray showed an absence of congruence of the glenohumeral joint upon abduction of the arm (Figure 1).

**Figure 2:**
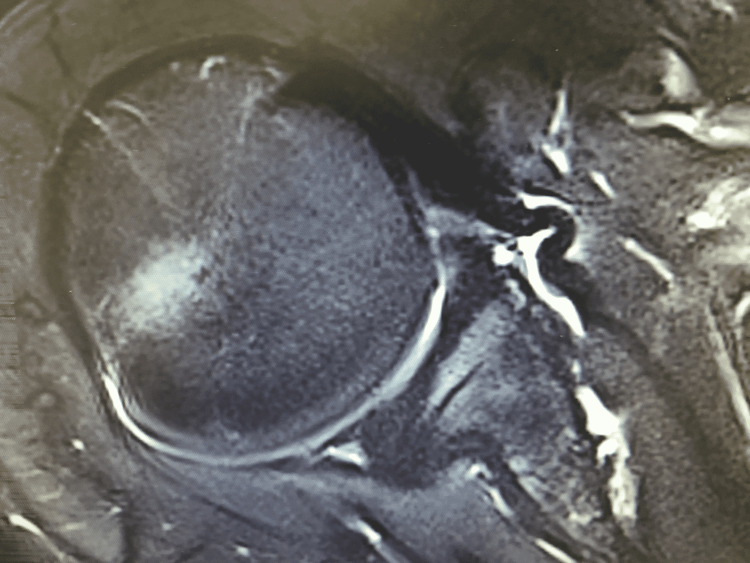
MRI scan showing a significant reverse Bankart lesion with a loose subscapularis tendon

**Figure 3:**
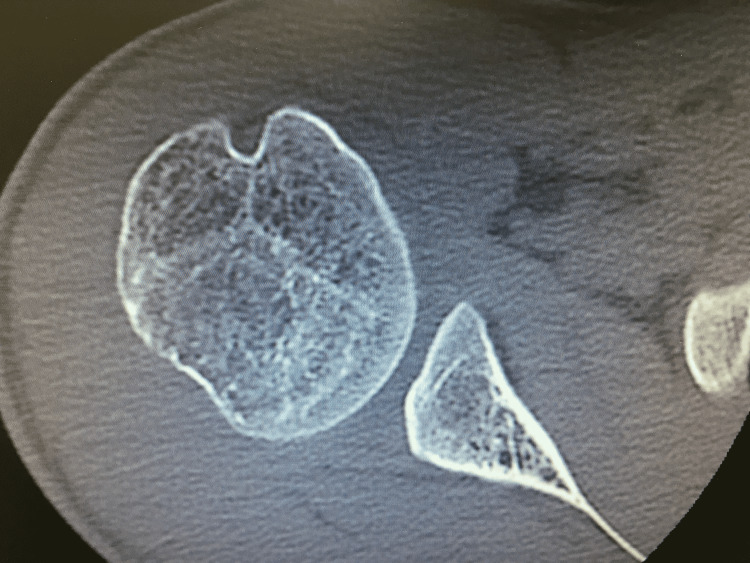
CT scan showing glenoid dysplasia with a loss of posteroinferior concavity with 28 degrees of retroversion

An MRI scan (Figure 2) showed a significant reverse Bankart lesion with a loose subscapularis tendon, most probably due to the multiple episodes of dislocation with relatively intact muscle fibers. A CT scan (Figure 3) showed reverse Hill-Sachs lesion on the humeral head, glenoid dysplasia with loss of concavity on the posteroinferior aspect, and 28 degrees of retroversion.

On the first operation, we performed a shoulder arthroscopy. The long head biceps tendon was found to be detached from its superior labrum insertion on the glenoid superior labrum from anterior to posterior (SLAP) lesion, so it was attached using one anchor. This procedure achieved a good stabilization effect on the humeral head so that it doesn’t dislocate posteriorly.

Then we decided to complete the planned intervention of glenoid osteotomy to increase stabilization; through a posterior approach, we developed the space between the middle and post chef of the deltoid muscle then between teres minor and infraspinatus muscles. We performed a capsulotomy and exposed the glenoid, and managed to correct the retroversion using osteotome by elevating the glenoid and impacting bone graft to improve the glenoid version, then we sutured the capsule to the labrum. The shoulder was immobilized for 15 days in a shoulder arm immobilizer.

**Figure 4:**
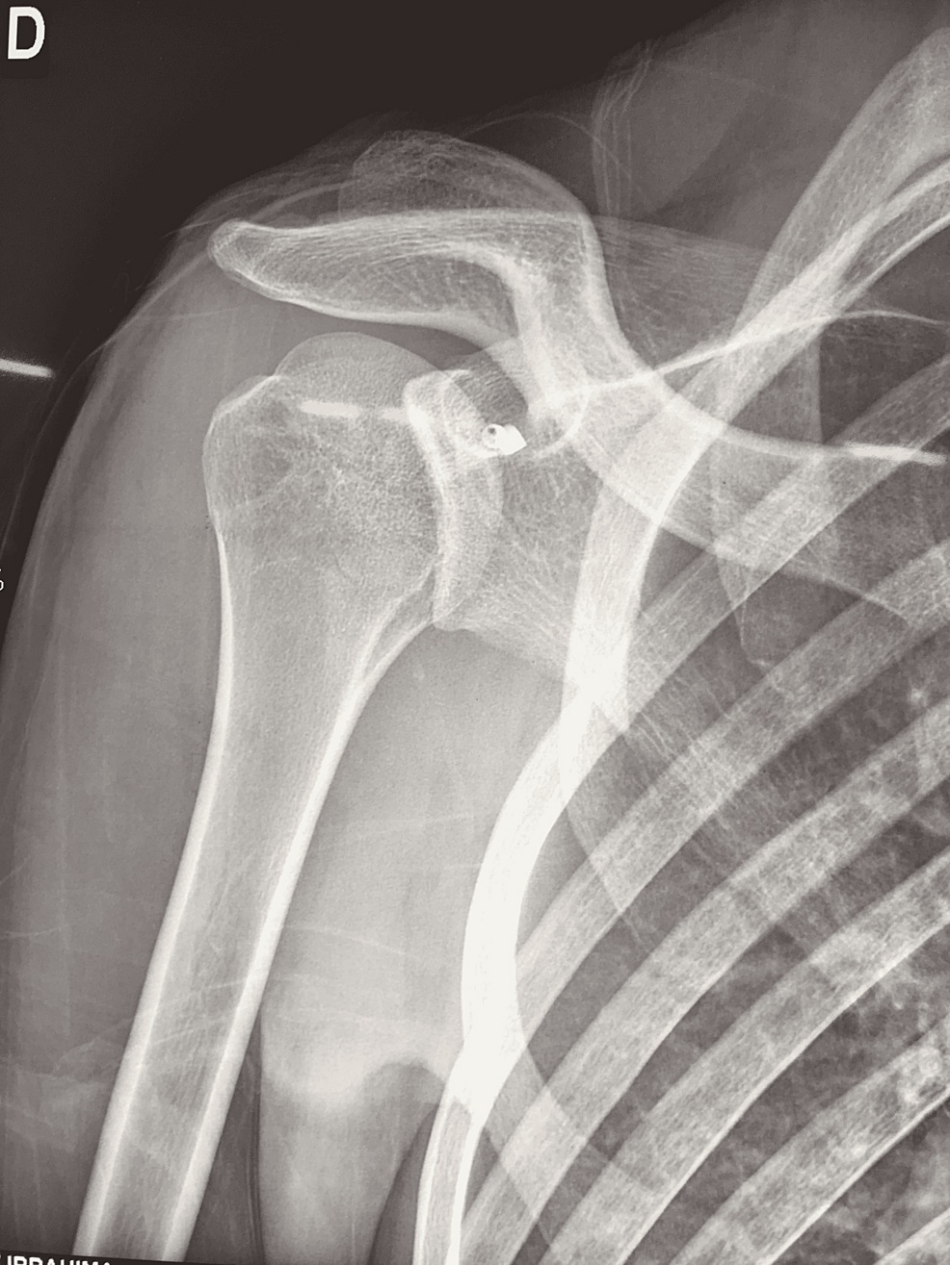
X-rays showing posterior dislocation of the right shoulder 15 days after the intervention.

The patient was seen 15 days after surgery, and the shoulder was dislocated posteriorly (Figure 4).

To resume, our patient was suffering from a neurological problem in his shoulder, causing muscle imbalance resulting in the eccentric force applied to the glenohumeral joint and dislocating the humeral head posteriorly. Thus, as a result of multiple episodes of dislocation, he developed structural damage in the glenoid and the humeral head, aggravating the situation and making the joint highly unstable.

Since the treatment of the posterior glenohumeral instability has to be individualized based on the patient´s injuries, medical history, clinical exam, and goals [7], after analyzing these factors, we concluded that the patient has several components of instability. It was dynamic and static, with both functional and structural pathological mechanisms as per ABC classification for posterior glenohumeral instability [8], and because the main cause of instability was functional (muscle imbalance and inability to keep the humeral head facing the glenoid), we decided to add further stabilization to the humeral head maintaining it in front of the glenoid without interfering with the rotation.

After induction of general anesthesia, the patient underwent a thorough examination under full relaxation assessing glenohumeral joint stability and humeral head translations. The shoulder was dislocating and reducing without difficulty.

**Figure 5:**
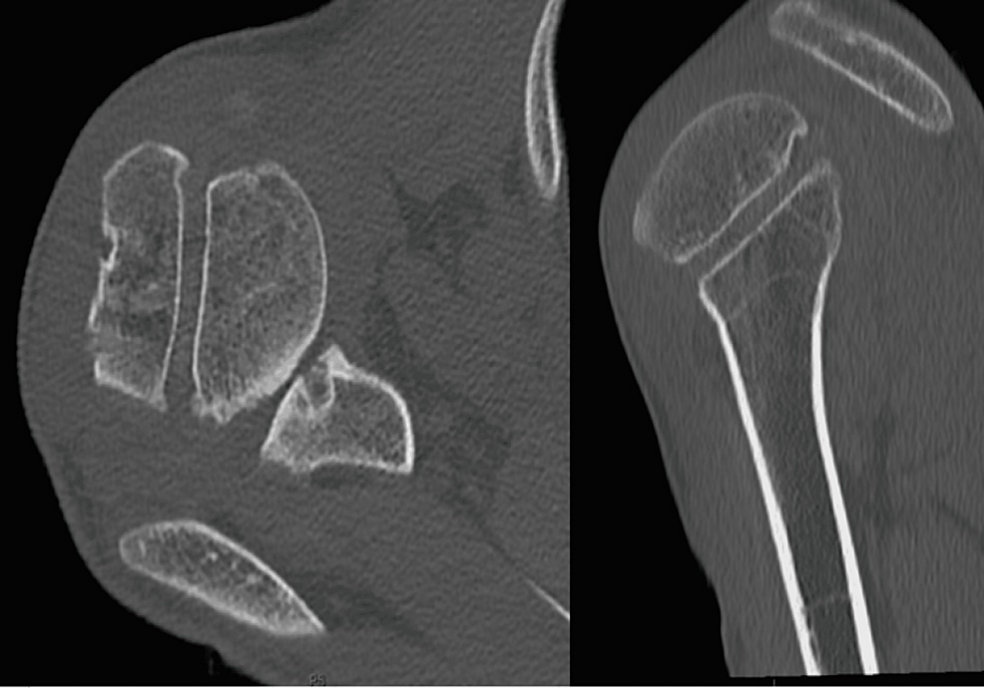
CT scan showing the tunnel drilled in the humeral head to pass the transplant

Following the examination under anesthesia, the patient was placed in a semi-sitting position, and the arm was positioned at approximately 50 degrees of forward flexion and abduction. We performed an open procedure for stabilization of the shoulder by replicating the coracohumeral ligament with a ligament advanced reinforcement system (LARS) transplant. We drilled a tunnel in the humeral head through which we passed the transplant (Figure 5).

**Figure 6:**
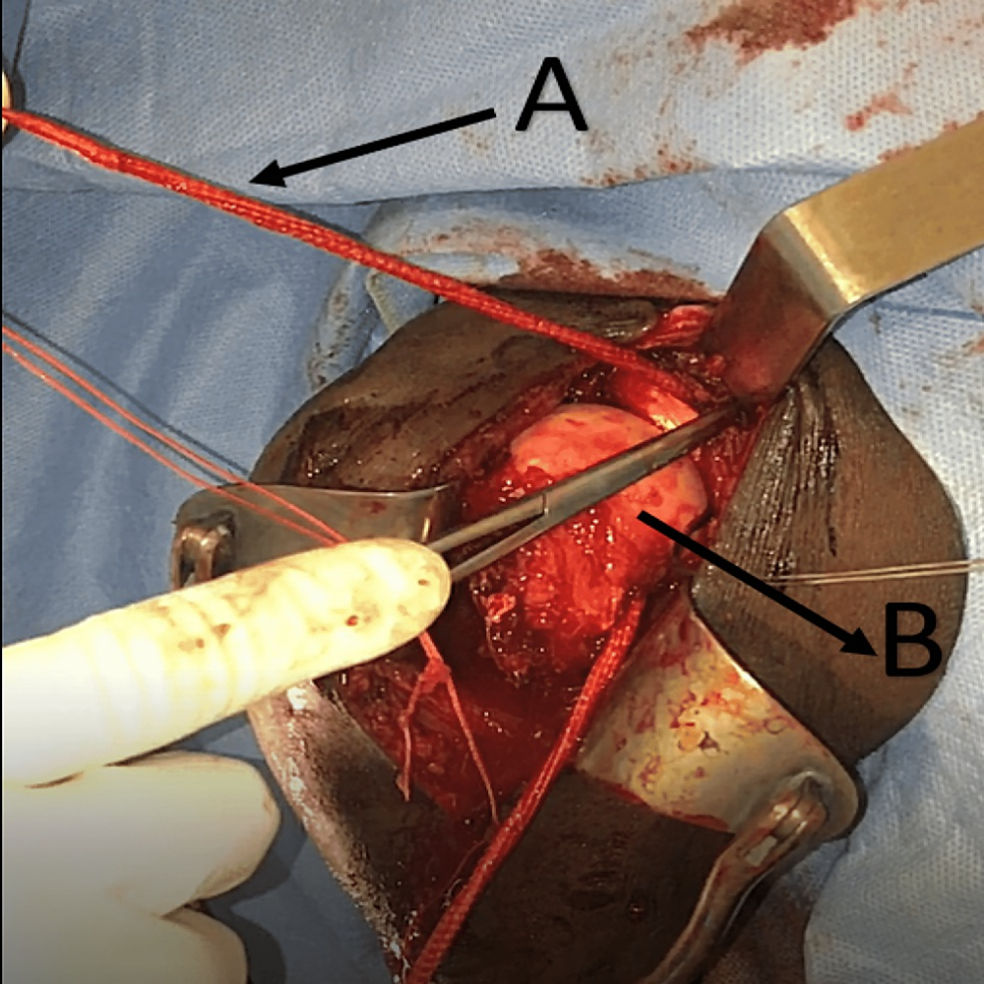
Image showing the LARS transplant passing through the humeral head drilled tunnel A) LARS transplant, B) humeral head LARS - ligament advanced reinforcement system

**Figure 7:**
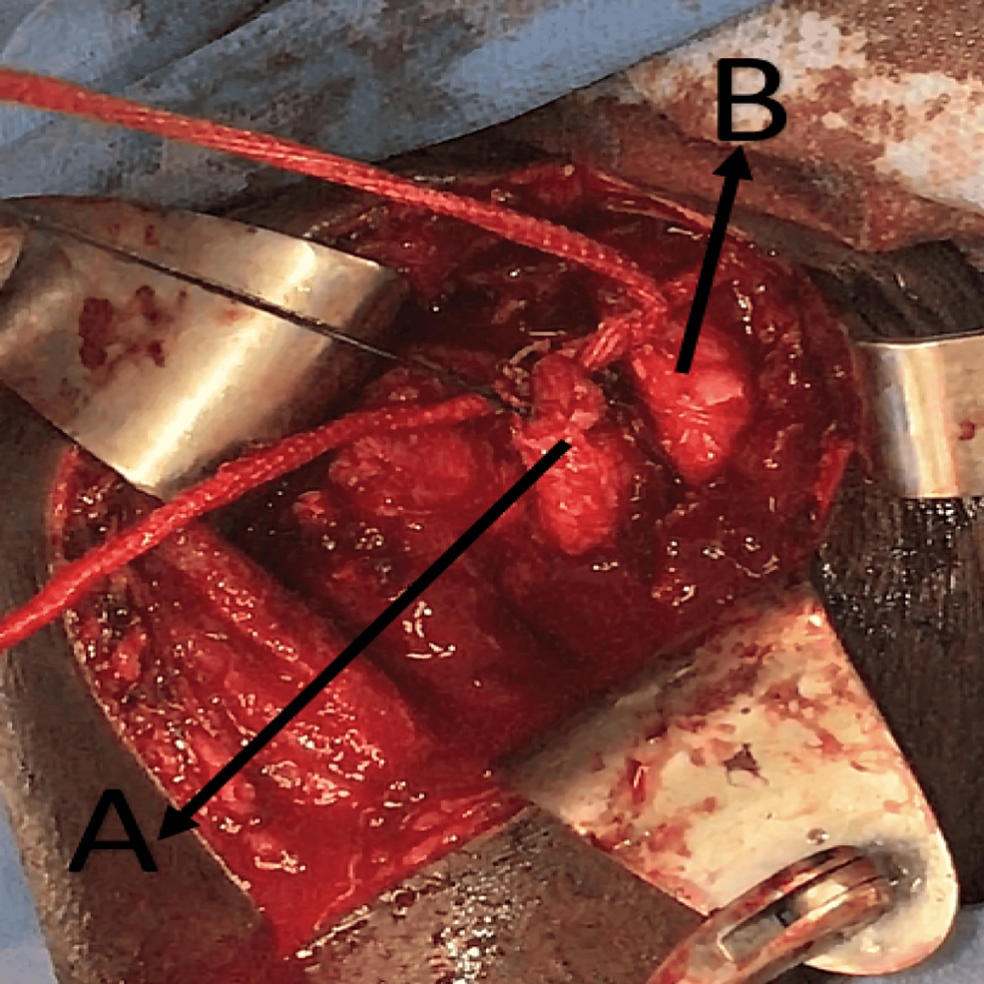
Image showing the LARS transplant tied around the conjoint tendon A) detached subscapularis tendon, B) conjoint tendon LARS - ligament advanced reinforcement system

The transplant was then pulled and tied around the conjoint tendon (short head of biceps tendon and coracobrachialis tendon) and stitched to itself (Figure 6, 7, Video 2)

**Video 2:** Video showing the transplant tied around the conjoint tendon and tightened preventing further translation of the humeral head with posteriorly directed force applied

**Figure 8:**
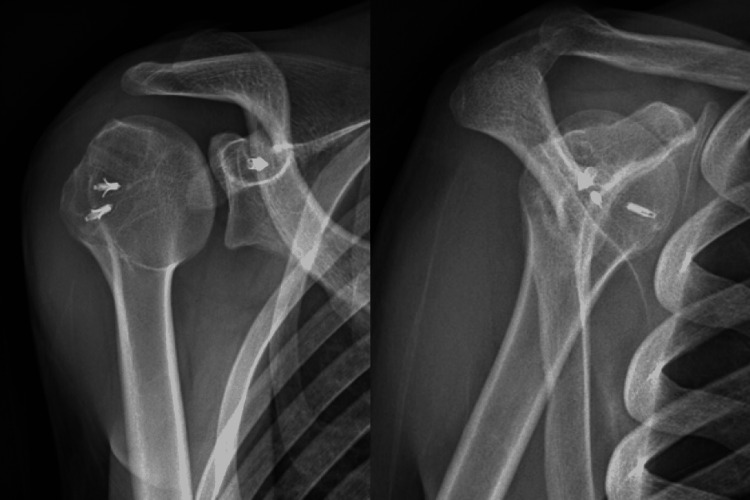
X-ray showing a congruent shoulder joint

The transplant had an excellent stabilizing effect with the complete impossibility for further posterior translation of the head, thus dislocation. The shoulder was tested peroperatively, showing good stability (Figure 8).

There was no compromise on the shoulder rotation. The loose subscapular tendon then was reattached to the humerus on the lesser tuberosity with two Mitek G2 anchors (DePuy Synthes Companies, Raynham, Massachusetts), and we immobilized the shoulder for six weeks to allow tissue healing.

At an average of 18 months after the operation, the X-ray showed congruent shoulder. The patient was relieved of the pain and was able to move his arm without any discomfort in different directions despite some limitations in range of motion: shoulder abduction of 90°, forward flexion of 120°, external rotation of 40°, and internal rotation of 55°.

He mentioned increased confidence with his shoulder during the movement of his arm without any episode of dislocation. The average relative score according to the system of Constant and Murley was 70 percent. The patient’s estimation of the value of the affected shoulder as a percentage of that of an entirely normal shoulder amount to 60% [3].

## Discussion

Shoulder instability is an excessive abnormal translation of the humeral head in relation to the glenoid. Multiple studies concluded that aberrant muscle activation patterns usually end up by a glenohumeral instability [9,10,11]. Common symptoms are pain upon range of motion, a loss of active movement due to weakness or blockage, in addition to a strong feeling of instability precluding a range of motion and restricting shoulder function.

The Stanmore classification of shoulder instability distinguishes the mechanism, pattern, and cause of instability and divides patients into three different groups [12]. Polar type I patients present with structural disturbance of the glenohumeral joint resulting from trauma. Polar type II patients usually present with inherent deficits such as capsular incompetency or altered concavity of the glenoid surface [13]. Polar type III describes a type of instability where the shoulder translates excessively, provoking a subluxation or dislocation every time the shoulder passes a particular phase of movement caused by an aberrant activation pattern of the rotator cuff and periscapular muscles.

Several surgical techniques have been described to treat a patient with posterior glenohumeral instability from posteroinferior capsular shift, to labral fixation, to glenoid osteotomy [14].

To the best of our knowledge, the management of such a complicated case of posterior shoulder instability has not been addressed in the literature. Modified Mclaughlin technique shows good results in a shoulder presenting reverse Hill-Sachs lesion as the main cause of recurrent dislocation, which is not present in our case. The posterior bone block procedure could have been a potentially acceptable option, but poor outcomes were found in patients with multidirectional instability with inevitable development of glenohumeral osteoarthritis [14].

Our patient had all these factors of instability because the aberrant muscle activation pattern due to his old minor cerebrovascular accident made the shoulder dislocates posteriorly indenting on the capsule, labrum, and the posterior glenoid, thus suppressing their stabilizing role. With every episode of dislocation, the subscapularis tendon was stretched more until it lost its function, so we performed these procedures to reproduce the static and dynamic stabilization of the shoulder, dealing with both functional and structural abnormalities. This case demonstrates that those procedures with proper physical therapy were successful in treating this complex form of shoulder instability with the ability to return to normal daily activity at 15 months.

## Conclusions

These interventions were sufficient to prevent the repetitive episodes of dislocation and ensure stabilization of a shoulder suffering from all three factors of instability: aberrant muscular activation pattern, capsular incompetency, and glenoid dysplasia. Following structured physical therapy, our patient had return to normal daily activities at 15 months.
